# A Systems' Biology Approach to Study MicroRNA-Mediated Gene Regulatory Networks

**DOI:** 10.1155/2013/703849

**Published:** 2013-11-17

**Authors:** Xin Lai, Animesh Bhattacharya, Ulf Schmitz, Manfred Kunz, Julio Vera, Olaf Wolkenhauer

**Affiliations:** ^1^Department of Systems Biology and Bioinformatics, University of Rostock, 18051 Rostock, Germany; ^2^Laboratory of Systems Tumor Immunology, Department of Dermatology, Faculty of Medicine, University of Erlangen-Nuremberg, Ulmenweg 18, 91054 Erlangen, Germany; ^3^Department of Dermatology, Venereology and Allergology, University of Leipzig, 04155 Leipzig, Germany; ^4^Institute for Advanced Study (STIAS), Wallenberg Research Centre at Stellenbosch University, Stellenbosch 7600, South Africa

## Abstract

MicroRNAs (miRNAs) are potent effectors in gene regulatory networks where aberrant miRNA expression can contribute to human diseases such as cancer. For a better understanding of the regulatory role of miRNAs in coordinating gene expression, we here present a systems biology approach combining data-driven modeling and model-driven experiments. Such an approach is characterized by an iterative process, including biological data acquisition and integration, network construction, mathematical modeling and experimental validation. To demonstrate the application of this approach, we adopt it to investigate mechanisms of collective repression on p21 by multiple miRNAs. We first construct a p21 regulatory network based on data from the literature and further expand it using algorithms that predict molecular interactions. Based on the network structure, a detailed mechanistic model is established and its parameter values are determined using data. Finally, the calibrated model is used to study the effect of different miRNA expression profiles and cooperative target regulation on p21 expression levels in different biological contexts.

## 1. Introduction

Although microRNAs (miRNAs) are physically small, they have been shown to play an important role in gene regulation [1]. Currently, an increasing number of studies are being carried out to deepen our understanding of miRNA regulatory mechanisms and functions. However, experimental approaches have limitations when dealing with complex biological systems composed of multiple layers of regulation such as the transcriptional and post-transcriptional regulation by transcription factors (TFs) and miRNAs [2]. Most experimental approaches focus on the identification of miRNA targets and the investigation of physiological consequences when perturbing miRNA expressions but are unsuited to provide a system-level interpretation for observed phenomena. Therefore, the introduction of a systematic approach, which can unravel the underlying mechanisms by which miRNAs exert their functions, becomes increasingly appealing.

The systems biology approach, combining data-driven modeling and model-driven experiments, provides a systematic and comprehensive perspective on the regulatory roles of miRNAs in gene regulatory networks [3–5]. To investigate a gene regulatory network, an iterative process of four steps is needed. (I) *Biological network construction*: a map is constructed that shows interactions among molecular entities (such as genes, proteins and RNAs), using information from literature and databases. (II) *Model construction*: depending on the biological problem investigated and experimental data available, the interaction map can be translated into a detailed mechanistic model that can simulate the temporal evolution of molecular entities. The values of parameters in this model can be determind from literature, databases or they are directly estimated from quantitative experimental data using optimization methods. (III) *Computational experiments*: once a model is established, it can be simulated and/or analyzed for its general behavior. (IV) *Experimental validation*: model predictions together with biological explanations are integrated to guide the design of new experiments, which in turn validate or falsify the model. If model predictions are in agreement with the experiments, the model justifies the biological hypotheses behind it. In turn, these hypotheses, which provide reasonable explanations for the biological phenomenon, lead to an enhanced understanding of the gene regulatory network. Otherwise, the structure of the mathematical model is modified to generate new hypotheses and suggest new experiments.

The application of the systems biology approach to the analysis of a gene regulatory network is demonstrated with a case study of the regulation of p21 by multiple miRNAs [4]. The network combining putative targets of TF and miRNA regulation with experimentally proven molecular interactions was constructed and visualized. Next, the network was translated into a detailed mechanistic model, which was characterized and validated with experimental data. Finally, the integration of quantitative data and modeling helped us to generate and validate hypotheses about mechanisms of collective miRNA repression on p21. 

## 2. Results

### 2.1. The Systems Biology Approach to Study miRNA-Mediated Gene Regulatory Networks

#### 2.1.1. Mathematical Modeling

The aim of our analysis is to unravel the complex mechanisms by which gene regulatory networks involving miRNAs are regulated. We iteratively integrate data from literature, experiments and biological databases into a detailed mechanistic model of a gene regulatory network. The model is then used to formulate and test hypotheses about mechanisms of miRNA target regulation and cellular process-related variability. The methodology includes four steps which are briefly summarized in Table 1. In the coming sections we present these steps in detail.

(*1)  Data Retrieval. *To construct a gene regulatory network composed by different levels of regulation, we collect information from different resources which are briefly described below, and more resources for data retrieval are introduced in Table 1.

- *Transcriptional level regulators*. Experimentally verified TFs for a gene can be extracted from literature or databases such as TRED, TRANSFAC, or HTRIdb [6–8]; the putative TFs, which are associated with conserved TF-binding sites residing in the promoter region of a gene, can for example, be extracted from the table of TFs with conserved binding sites in the UCSC genome browser or the TRANSFAC database [9]. miRGen 2.0 is a database that provides both predicted and experimentally verified information about miRNA regulation by TFs [10].
- *Post-transcriptional regulators*. Databases such as miRecords, Tarbase and miRTarBase are repositories of experimentally validated miRNA:gene interactions [11–13]. Predictions of miRNA:gene interactions are accumulated in databases like miRWalk [14].
- *Protein-protein interactions*. Both the Human Protein Reference Database (HPRD) [15] and the STRING database [16] document experimentally verified protein-protein interactions; besides, STRING also provides putative protein-protein interactions ranked by confidence scores. Further details about the exact mechanism of protein-protein interactions can be found in Reactome [17].

(*2)  Network Construction and Visualization.* Based on the information collected, a gene regulatory network is constructed and visualized for providing an overview. For this purpose, we recommend CellDesigner which uses standardized symbols (Systems Biology Graphical Notation—SBGN) [18] for visualization and stores gene regulatory networks in the SBML format (Systems Biology Markup Language) [19]. CellDesigner also provides the possibility to simulate temporal dynamics of the gene regulatory network due to the integration of the SBML ODE (ordinary differential equation) solver. Besides, Cytoscape is another powerful tool for integration of biological networks and gene expression data [20]. 

For assessing the reliability of interactions considered in gene regulatory networks, confidence scores can be computed as being documented in our previous publication [4]. The factors that are used to determine the confidence score for molecular interactions can be: the number of publications reporting an interaction, experimental methods used to identify an interaction, interaction types and computational predictions. The computed confidence scores range from 0 to 1, where values towards 1 indicate higher confidence, whereas values towards 0 indicate lower confidence in a given interaction. For example, the confidence score for a miRNA:gene interaction can be calculated using the following equation:

(1) $$\begin{array}{c} \;\;S_{\text{miRNA}:\text{gene}}=\frac{w_{p}\cdot S_{p}+w_{m}\cdot S_{m}+w_{\text{bs}}\cdot S_{\text{bs}}}{w_{p}+w_{m}+w_{\text{bs}}}, \end{array}$$

where *w*_〈*p*,*m*,bs〉_ are weights that are assigned to the scores which account for the number of publications (*S*_*p*_), detection method (*S*_*m*_) and the number of predicted binding sites (*S*_bs_). The values of the weights can be assigned based on expert knowledge, and the higher the value of the weight is, the bigger impact it has on the confidence score for the interaction. The values of *S*_*p*_ and *S*_bs_ can be calculated using the logarithmic equation: *S*_〈*p*,bs〉_(*n*) = log⁡_*m*+1_(*n*), where *n* denotes the number of publications describing the miRNA:gene interaction or the number of binding sites that the miRNA has in the 3′ UTR (untranslated region) of the gene. The value of *m* is a cut-off that represents the number of publications or binding sites required for *S*_〈*p*,bs〉_ to obtaining their maximum values. Various methods such as western blots, qRT-PCR and reporter assays can be applied to support the miRNA:gene interaction, but these methods provide experimentalists with different levels of confidence, thus differing confidences can be reflected using different values for *S*_*m*_ based on the experience of experimentalists. Of note, although the confidence scores cannot be directly converted into a mathematical model, with the help of these scores we can discard non-reliable putative interactions to generate the ultimate version of a gene regulatory network. The final version of the network can be further analyzed to identify regulatory motifs like feedforward loops (FFLs), for example, with the help of the Cytoscape plugin NetDS [21]. Thereafter, the complete network or parts of it can be converted into a detailed mechanistic model which is described in detail in the following section. 

(*3)  Model Construction and Calibration*. After the construction, visualization and refinement of a gene regulatory network, it is converted into a detailed mechanistic model which enables the investigation of unanswered biological questions and validation of hypotheses. Ordinary differential equations (ODEs) describe how processes of synthesis, biochemical modification and/or degradation affect the temporal concentration profile of biochemical species like proteins, RNAs and metabolites. An ODE model can be constructed using appropriate kinetic laws such as the law of mass-action, which states that the rate of a chemical reaction is proportional to the probability that the reacting species collide. This collision probability is in turn proportional to the concentration of the reactants [22]. A general representation of ODE-based models using mass-action kinetics is given by the following equation

(2) $$\begin{array}{c} \frac{dx_{i}}{dt}=\overset{m}{\underset{\mu =1}{\sum }}c_{i\mu }\cdot k_{\mu }\cdot \overset{n}{\underset{j=1}{\prod }}{x_{j}}^{g_{\mu j}},\;i\in \left\{1,2,\ldots ,n\right\}, \end{array}$$

where *x*_*i*_ represents state variables which denote the molar concentration of the *i*th biochemical specie. Every biochemical reaction  *μ*  is described as a product of a rate constant (*k*_*μ*_) and biochemical species (*x*_*j*_, *j* ∈ {1,2,…, *n*}) that are involved in this reaction. *c*_*iμ*_, the so-called stoichiometric coefficients, relate the number of reactant molecules consumed to the number of product molecules generated in the reaction *μ*. *g*_*μj*_ denotes kinetic orders which are equal to the number of species of *x*_*i*_ involved in the biochemical reaction  *μ*.  The rate constants, kinetic orders and the initial conditions of state variables are defined as model parameters. Besides mass-action kinetics, other kinetic rate laws such as Michaelis-Menten kinetics, Hill equation and power-laws are also frequently used in ODE models.

After ODEs are formulated, the model requires to be calibrated, a process by which parameter values are adjusted in order to make model simulations match experimental observations as good as possible. To do so, there are two possible means: characterization of parameter values using available biological information or estimation of parameter values using optimization methods. Some parameter values can be directly measured or obtained from literature or databases. For example, the half-life (*t*_1/2_) of some molecules (e.g., protein) can be measured *in vitro* via western blotting. This information can be used to characterize their degradation rate constants through the equation *k*_deg⁡_ = (ln⁡ 2/*t*_1/2_). The database SABIO-RK provides a platform for modelers of biochemical networks to assemble information about reactions and kinetic constants [27]. However, for most model parameters, whose values cannot be measured in laboratories or be accessed from literature or databases, parameter estimation is a necessary process to characterize their values. Before running parameter estimation, initial parameter values and boundaries should be set within physically plausible ranges. To do so, the database BioNumbers provides modelers with key numbers in molecular and cell biology, ranging from cell sizes to metabolite concentrations, from reaction rates to generation times, from genome sizes to the number of mitochondria in a cell [28]. After parameter estimation, unknown parameter values are determined using optimization methods which can minimize a cost function that measures the goodness of fit of the model with respect to given quantitative experimental data sets. Parameter estimation using optimization algorithms is an open research field, in which several methods have been developed according to the nature and numerical properties of biological data analyzed. The discussion for choosing proper optimization methods for parameter estimation is beyond the scope of this paper, but the interested reader is referred to the paper published by Chou and Voit [29]. 

(*4)  Model Validation and Analysis*. Usually, the model simulations are compared with the experimental data used for the parameter estimation, but a good agreement between both is not enough to guarantee the predictive ability of the model. Therefore, it is necessary to validate the model with data sets that are not used during the parameter estimation. This process is called model validation and can ensure more reliable and accurate model predictions. To do so, the data generated in new experiments or extracted from literature are compared with model simulations, which are obtained after configuring the model according to the new experimental settings. Once a model is validated, it can be used to perform predictive simulations, which are helpful to study the dynamic properties of biochemical systems, guide the design of new experiments in the laboratory and formulate additional hypotheses. In addition, tools such as sensitivity and bifurcation analysis can be used to study complex properties and behavior of the modeled system. Sensitivity analysis is used to evaluate the influence of model parameters (e.g., initial concentrations of the state variables and rate constants) on model outputs, such as the temporal behavior of network components [30]. Bifurcation analysis is employed to detect control parameters (also known as bifurcation parameters) whose variations are able to change drastically the dynamical properties of the biochemical system, as well as the stability of its fixed points [31]. The application of these tools to mathematical modeling is beyond the scope of this paper, but the interested reader is referred to the publication of Zhou et al. [32] and Marino et al. [33]. 

#### 2.1.2. Experiment Methods

As mentioned in the previous sections, after a model is established, it can be calibrated and validated using temporal experimental data. To do so, the data can either be derived from literature or generated to calibrate the model by own experiments. In case of ODE models, the most suitable data for model calibration is quantitative time-series data obtained from perturbation or quantitative dose-response experiments. The experiments, in which time-series data are measured for different regulators (such as miRNAs and TFs) of a gene regulatory network, can be obtained using the techniques described in the subsections below. 

(*1)  qRT-PCR*. Quantitative real time polymerase chain reaction (qRT-PCR) has been used to identify mRNAs regulated by overexpression or silencing of a specific miRNA [34, 35]. miRNAs typically exhibit their regulatory effects by associating with specific 3′ UTR regions of the mRNAs called miRNA seed regions [34]. This association can lead either to a temporary inhibition of translation or complete degradation of the mRNA in which case qRT-PCR mediated detection is beneficial. For this, the cells are transfected with the miRNA and non-targeting control oligonucleotides at an appropriate concentration using either a lipid based transfection reagent or nucleofection. The transfected cells are then incubated for the necessary time periods (e.g., 24 h, 48 h, 72 h, etc.) after which lysates are prepared and total RNA is extracted. 1000–2000 ng of the RNA is then converted into cDNA using a reverse transcription kit. Taqman qRT-PCR is performed using 10–20 ng of cDNA and primers labeled with fluorescence probes to detect the transcriptional levels of the target mRNA. Housekeeping genes like GAPDH and HPRT are used as endogenous controls for data normalization. Relative expression at different time points is determined by comparing the Ct values of the miRNA transfected cells with Ct values of non-targeting control transfected cells and expressed as relative expression [36]. However, qRT-PCR in spite of being highly sensitive is applicable specifically under conditions of complete or partial degradation of the target mRNA. miRNA-mediated inhibition in translation can be better demonstrated by techniques like immunoblotting. 

(*2)  Western Blot/Immunblotting*. Western blotting or protein immunoblotting is a technique to detect the expression of a gene at protein level. This technique is particularly useful in determining the regulatory effects of a miRNA on expression of a target gene which is temporarily inhibited. For this the cells are transfected with a miRNA or an antagomiR as mentioned above and cell lysates are prepared at appropriate time points using protein lysis buffers (e.g., Radio-Immunoprecipitation Assay buffer or RIPA) containing lysis agents like Dithiothreitol (DTT) and protease inhibitors. The proteins from each sample are quantitated using Bradford or BCA reagents and compared with bovine serum albumin (BSA) standards for accurate protein estimation. 20–40 *μ*g of protein is then loaded and resolved on a sodium dodecyl sulfate polyacrylamide gel (SDS-PAGE) along with a pre-stained protein marker. The protein bands are then transferred onto a nitrocellulose membrane followed by incubation with the appropriate primary and secondary antibodies linked to fluorescent dyes or horse radish peroxidase enzyme (HRP). The protein expression is then analyzed using either fluorescence or chemiluminiscence HRP substrates in gel documentation systems (e.g., LI-COR Odyssey). Housekeeping genes like *β*-actin or *β*-tubulin are used for protein normalization. The time point for maximum target gene suppression generally varies depending on the number of miRNA binding sites at the 3′ UTR of the target gene and the extent of complementarity of the seed region [37]. Immunoblotting is a widely used technique to provide confirmatory evidence for the inhibitory effects of miRNA at the protein level, but it fails to explain the underlying interaction mechanisms. 

(*3)  Reporter Gene Assay*. As each miRNA can inhibit the expression of a large number of genes, regulation of a particular target gene may either be by direct interaction or be an indirect consequence of it. In direct regulation, a miRNA binds to the complementary sequences at the 3′ UTR of a target gene and thereby suppresses its expression. As a consequence of this, the expression levels of a number of downstream genes (indirect targets) are also dysregulated making it crucial to differentiate between primary and secondary miRNA targets. To determine the interaction specificity, a reporter construct (luciferase) with intact or mutated 3′ UTR of the target gene cloned at the 5′ end is co-transfected into the cells along with the miRNA. The regulatory effect of the miRNA on the target gene expression is then measured using the expression of a reporter gene. In the absence of the appropriate binding sequences (mutated 3′ UTR), the miRNA cannot suppress the reporter mRNA suggesting that the suppressive effect of the miRNA is mediated by a direct interaction. The reporter activity can be analyzed at different time points such as 24 hr, 48 hr and 72 hr to determine the time dependent suppression of a target gene expression by a miRNA. 

### 2.2. Case Study: The Regulation of p21 by Multiple and Cooperative miRNAs

#### 2.2.1. Construction and Visualization of p21 Regulatory Network

By using the approach described above, we investigated the regulation of p21 by its multiple targeting miRNAs. p21, also known as cyclin-dependent kinase inhibitor 1 (CDKN1A), is a transcriptional target of p53. It is required for proper cell cycle progression and plays a role in cell death, DNA repair, senescence and aging (reviewed in [38]). Interestingly, p21 was the first experimentally validated miRNA target hub, which is a gene that is simultaneously regulated by many miRNAs. This made it an ideal candidate for a case study of our approach [23]. To do so, we first constructed a p21 regulatory network using the following steps:

- We extracted miRNA-target interactions from the publication of Wu et al. [23] where a list of predicted p21-regulating miRNAs was subjected to experimental validation.
- Experimentally verified TFs of p21 were extracted from literature and putative TFs having conserved binding sites in the 5 kb upstream region of the p21 open reading frame were extracted from UCSC table browser. A list of TFs controlling the expression of the miRNAs was constructed using information of experimentally proven TF-miRNA interactions extracted from TransmiR (release 1.0) [39]. In addition, we generated a list of putative TFs of miRNAs with binding sites in the 10 kb upstream region of the miRNA genes using information from the databases PuTmiR (release 1.0) [40] and MIR@NT@N (version 1.2.1) [41], and from the table of TFs with conserved binding sites in the UCSC genome browser (hg18) [9].
- Information about protein interactions was extracted from the Human Protein Reference Database (HPRD, release 9.0) [15] and the STRING database (release 9.0) [16]. Only the experimentally verified p21-protein interactions were used to construct the network.
- Additionally, we associated the TFs in the network to nine biological processes based on the Gene Ontology (GO) [42]. The corresponding GO terms were cell proliferation, cell apoptosis, immune response, inflammatory response, cell cycle, DNA damage, cell senescence, DNA repair and cell migration.

**Figure 1:**
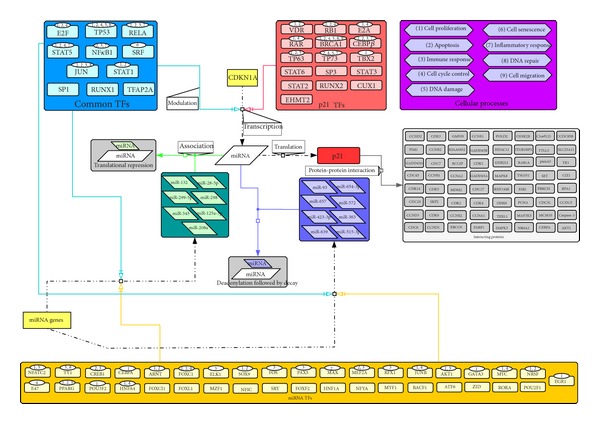
p21 regulatory network. The network contains several layers of regulators of p21: TFs (light blue and red boxes), miRNAs (dark blue and green boxes), and proteins (grey boxes). In each big box, there are small boxes which represent individual components of this layer of regulation. miRNAs are classified into two groups according to the mechanisms by which the expression of p21 is repressed. One group causes p21 translation repression (green box). These miRNAs bind to p21 mRNA resulting in the repressed translation of p21 but unchanged mRNA expression level. The other group of miRNAs (dark blue box) decreases the stability of p21 mRNA by modifying its structure, leading to mRNA decay and finally the downregulation of p21. TFs are classified into three groups: p21 TFs (red), miRNA TFs (yellow) and their common TFs (light blue). The p21 interacting-proteins are framed in the grey boxes. The purple boxes represent nine processes, and the TFs associated with these processes are indicated in the ellipses above them using corresponding figures. This data is adapted from our previous publication [4].

Next, we visualized the network in CellDesigner and computed a confidence score for each interaction in the network (Figure 1). The confidence scores provide us with the reliability of the interactions considered in p21 regulatory network. With the help of these scores, we discarded non-reliable interactions and constructed the mechanistic model accounting for p21 regulation by its targeting miRNAs. Besides, the interested experimentalists can further use this information to choose reliable interacting candidates of p21 for designing relevant experiments. The scores for each interaction of p21 regulatory network are shown in Table 2. 

#### 2.2.2. Mechanistic Modeling of p21 Regulation by Its Targeting miRNAs

(*1)  Model Construction*. After constructing the regulatory network, a detailed mechanistic model of ODEs, which describes the biochemical reactions underlying the regulation of p21 was established. We chose the ODE modeling approach, as it is a simple formalism for describing temporal dynamics of biochemical systems and a wide range of tools are available to explore their properties. Precisely, the model considered the mRNA (mp21; (3)) and protein (p21; (6)) of the miRNA target hub p21, the p21-targeting miRNAs (miR_*i*_;  *i* ∈ (1,…, 15); (5)), and the complexes formed by p21 mRNA and miRNA, [mp21 | miR_*i*_] (4). Altogether, the model is constituted by 32 state variables and 64 parameters:

(3)  dmp21dt=ksynmp21·fact(TFmp21)−mp21·(kdeg⁡mp21+∑ikasscomplexi·miRi),

(4) d[mp21 ∣ miRi]dt =kasscomplexi·mp21·miRi−kdeg⁡complexi·[mp21 ∣ miRi],

(5) dmiRidt=ksynmiRi·fact(TFmiRi)−miRi·(kdeg⁡miRi+kasscomplexi·mp21),

(6)  dp21dt=ksynp21·mp21+kdeg⁡p21·p21,

(7)   mp21Total=mp21+∑i[mp21 ∣ miRi].

For mp21, processes considered in the model were: (i) its synthesis (*k*_syn_^mp21^) mediated by TFs (*f*_act_(TF_mp21_)), (ii) its degradation (*k*_deg⁡_^mp21^), and (iii) its association with a miRNA (*k*_ass_^complex_*i*_^). For each miR_*i*_, processes considered were: (i) the synthesis (*k*_syn_^miR_*i*_^) mediated by TFs (*f*_act_(TF_miR_*i*__)), (ii) the degradation (*k*_deg⁡_^miR_*i*_^), and (iii) the association with the p21 mRNA target (*k*_ass_^complex_*i*_^). For each [mp21 | miR_*i*_] complex, processes considered were: (i) the formation of the complex by a miR_*i*_ and the p21 mRNA (*k*_ass_^complex_*i*_^), and (ii) the complex degradation (*k*_deg⁡_^complex_*i*_^). For p21, processes considered were: (i) its synthesis (*k*_syn_^p21^), and (ii) its degradation (*k*_deg⁡_^p21^). An additional algebraic equation accounting for the total measurable amount of p21 mRNA (mp21_Total_) was also included. The SBML file of the model is available for download at http://www.sbi.uni-rostock.de/uploads/tx_templavoila/p21_TargetHub_03092013.xml. 

(*2)  Model Calibration and Validation*. For model calibration, we fixed some parameter values using published data and estimated the other unknown parameters using the time-series data published by Wu et al. [23], in which the p21 mRNA (northern plot) and protein levels (western plot) were measured 48 hr after transfection of individual p21-targeting miRNAs into human embryonic kidney 293 cells. The unknown parameter values were estimated using an iterative method combining global (particle swarm pattern search) [43] and local (downhill simplex method in multi-dimensions) [44] optimization algorithms. For each miR_*i*_ considered in the model, the method minimizes the distance between model simulations and experimental data using the following cost function

**Figure 2:**
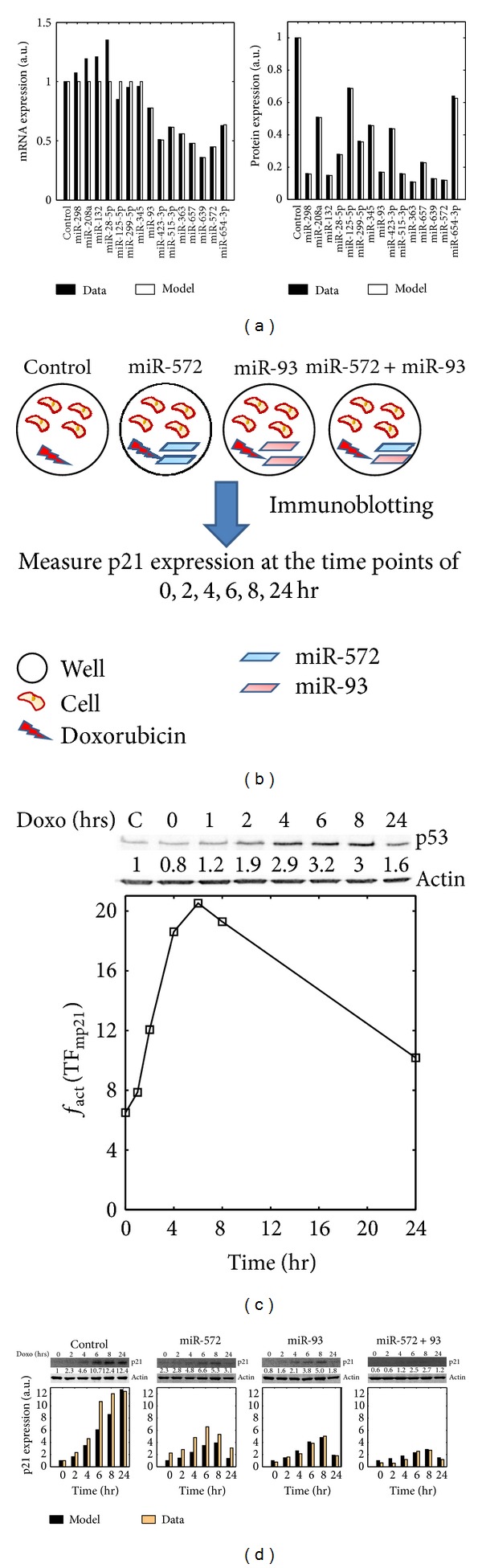
Model calibration and validation. (a) Model calibration. The figures show the relative change of the p21 mRNA and protein expression levels after overexpression of the indicated miRNAs (Model: model simulation; Data: experimental data). These data were normalized to the control group in which the p21 mRNA and protein expression levels were measured when the miRNAs were normally expressed (a.u.: arbitrary unit). (b) Experimental workflow. In the experiments, Sk-Mel-147 cells were seeded in six well plates. Then, mature miRNA mimics were transfected individually at a concentration of 100 nM (miR-572 and miR-93) or in combination at 50 nM each (miR-572 + miR-93). After 48 hr transfection with miRNA mimics, the cells were pulse treated with 250 nM doxorubicin for 1 hour after which normal growth medium was replenished. The immunblotting were performed to measure p21 expression at 0, 2, 4, 6, 8 and 24 hr post-doxorubicin treatment. (c) Temporal dynamics of p21 transcriptional function. Afterdoxorubicin treatment, the expression of p53, a TF of p21, was measured using immunblotting and these data were used to characterize the transcriptional function of p21 using MATLAB linear interpolation function. (d) Model validation. We measured the expression of p21 protein in response to genotoxic stress in the four scenarios as described in the main text. The measured data (Data) were compared with the model simulations (Model). The figures (a), (c) and (d) are adapted from our previous publication [4].

(8) FcostmiRi=[mp21simmiRi(t)−mp21exp⁡miRi(t)]2(δimp21)2+[p21simmiRi(t)−p21exp⁡miRi(t)]2(δip21)2i∈[1,…,15],

where  mp21_sim  _^miR_*i*_^(*t*)  and  mp21_exp⁡_^miR_*i*_^(*t*)  represent the simulated p21 mRNA and protein expression levels for each miR_*i*_ at time point *t*.  p21_sim_^miR_*i*_^(*t*)  and  p21_exp⁡_^miR_*i*_^(*t*)  represent the measured value for each miR_*i*_ at time point *t*, and their standard deviations are  *δ*_*i*_^mp21^and  *δ*_*i*_^p21^. Here, *t* is the time point (48 hr) after overexpression of the individual miRNAs in embryonic kidney 293 cells at which the expression levels of the p21 and its mRNA were measured [23]. The model calibration results are shown in Figure 2(a) and the obtained parameter values are listed in Table 3.

Experimental results showed that a stronger repression of the target gene can occur when two miRNA binding sites on the target mRNA are in close proximity [24, 45]. To test the consequences of this hypothesis, we predicted cooperative miRNA pairs for p21, with seed site distances between 13–35 nt in the p21 3′ UTR. To substantiate the cooperative effect associated with pairs of miRNAs, we introduced a group of new state variables ([mp21 | miR_*i*_ | miR_*i*_]) into the original model. These state variables account for the ternary complexes composed of p21 mRNA and two putatively cooperating miRNAs (miR_*i*_ and miR_*j*_). For these new variables, processes considered are: (i) the association of p21 mRNA with miR_*i*_ and miR_*j*_ into a complex (*k*_ass_^co-complex_*i*,*j*_^), and (ii) the degradation of the complex (*k*_deg⁡_^co-complex_*i*,*j*_^). After expansion, the corresponding modified and new ODEs are listed below:

(9) dmp21dt=ksynmp21·fact(TFmp21)−mp21·(kdeg⁡mp21+∑ikasscomplexi·miRi +∑i,jkassco-complexi,j·miRi·miRj),

(10) d[mp21 ∣ miRi ∣ miRj]dt =kassco-complexi,j·mp21·miRi·miRj  −kdeg⁡co-complexi,j·[mp21 ∣ miRi ∣ miRj].

To model stronger repression of the target gene by cooperating miRNAs, we assumed a stronger association rate constant for the complex [mp21 | miR_*i*_ | miR_*j*_] which is equal to the sum of their individual association rate constants (*k*_ass_^co-complex_*i*,*j*_^ = *k*_ass_^complex_*i*_^ + *k*_ass_^complex_*j*_^). Similarly, the degradation rates of the complexes [mp21 | miR_*i*_ | miR_*j*_] were assumed to be equal to the sum of degradation rate constants of single miRNA binding complexes (*k*_deg⁡_^co-complex_*i*,*j*_^ = *k*_deg⁡_^complex_*i*_^ + *k*_deg⁡_^complex_*j*_^). However, it has to be noted that these added equations are an abstract description of miRNA cooperativity, because the details of this mechanism are not yet known.

To experimentally validate the capability of our model to predict the relative p21 concentrations regulated by cooperative miRNAs, we selected miR-572 and miR-93 as a case study. These two miRNAs were chosen, because their predicted target sites in the p21 3′ UTR are in close proximity to each other and thereby, they can induce cooperative repression on p21 as suggested in [24]. The experiments were performed as follows:

- Melanoma cells (Sk-Mel-147) were transfected with the mature miRNA mimics of the two miRNAs either individually (100 nM) or in combination (50 nM each), whereas untreated cells were used as control (Figure 2(b)).
- Next, the cells were treated with doxorubicin, a genotoxic-stress inducing agent. The agent can upregulate the expression of p53, which is a known TF of p21, and therefore it can result in the upregulation of p21 (Figure 2(c)).
- After doxorubicin treatment, the expression levels of p21 were measured by immunoblotting at different time points (0, 2, 4, 6, 8, 24 hr). The p21 expression values were normalized based on the p21 expression level in the control group measured at time point 0 hr (Figure 2(d)).

**Figure 3:**
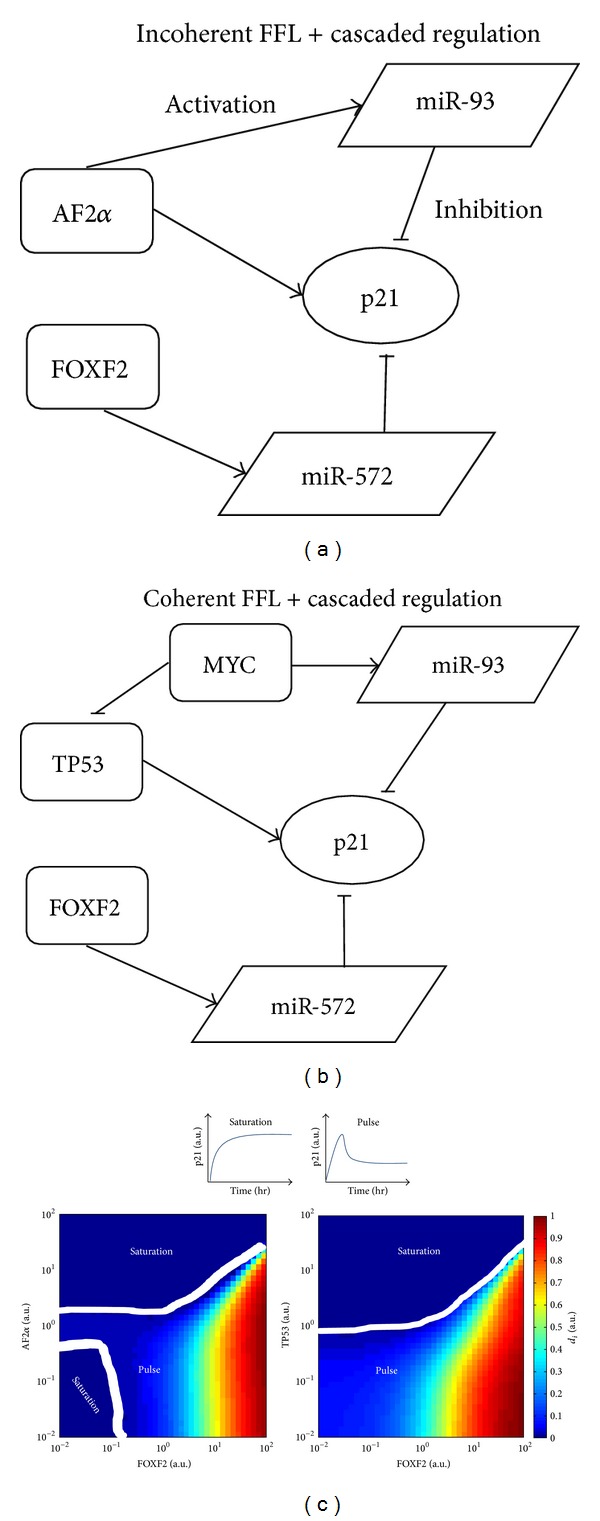
Different p21 dynamics in different network motifs. We ran simulations to show the different dynamics of p21 for two different network motifs (a) and (b). Through simulations, two dynamical patterns of p21 were identified: saturation and pulse ((c), top). For each network motif, the corresponding distributions of the two dynamical patterns were plotted ((c), bottom). For different combinations of the transcriptional strengths, the normalized distance (*d*_*i*_) between peaks (*p*_*i*_) and steady states (ss_*i*_) of p21 is determined by the equation *d*_*i*_ = (*p*_*i*_ − ss_*i*_)/*p*_max⁡_, *p*_max⁡_ = max⁡⁡(*p*_1_,…, *p*_*n*_). If *d*_*i*_ = 0, for the corresponding combination of transcriptional strengths the p21 dynamics is saturation, otherwise it is pulse. The regions showing different dynamical patterns of p21 are separated using the white lines.

By doing so, we obtained the p21 response after genotoxic stress in four scenarios: (1) endogenous miRNA expression (Control); (2) overexpression of miR-572; (3) overexpression of miR-93; and (4) both miRNAs moderately overexpressed. Thereafter, we derived a model of seven ODEs based on the original equations which was configured according to the designed experiments, making the simulation results comparable with the experimental data. As shown in the Figure 2(c), the simulations are in good agreement with the experimental observations. The individual overexpression of miR-572 or miR-93 led to the reduction of the upregulation of p21 response after genotoxic stress induction. The two miRNAs cause different degrees of repression due to their different repression efficiencies on p21. Interestingly, the combined overexpression of both miRNAs induced the strongest downregulation of p21, and therefore verifying the hypothesis of their cooperative regulation of p21. Above all, the results not only validated the model but also demonstrated the ability of our method to identify cooperative miRNA pairs for p21. 

(*3)  Predictive Simulations*. As there are abundant of network motifs such as FFLs in p21 regulatory network and these network motifs are important for determining p21 dynamics, it is interesting to investigate the dynamics of p21 in network modules where FFLs are involved. To do so, two network modules including both miR-93 and miR-572, and their TFs were exemplified. In Figure 3(a), the network module contains an incoherent FFL composed by AF2*α*, miR-93 and p21, and a cascaded regulation in which p21 is repressed by FOXF2 via miR-572; in Figure 3(b), the same cascaded regulation together with a coherent FFL composed of TP53, MYC, miR-93 and p21 forms another regulatory module of p21. By modulating the transcriptional strengths of the two miRNAs by their TFs, two types of p21 dynamics were identified: saturation and pulse (Figure 3(c), top). In the former, the p21 expression increases and reaches its steady state at the highest level; in contrast, in the latter the p21 expression increases to a peak and thereafter drops to a steady state at lower level. For the two different network modules, various combinations of transcriptional strengths of the two miRNAs lead to different distributions of the two p21 dynamical patterns. For the network module of an incoherent FFL plus the cascaded regulation (Figure 3(c), bottom left), the saturation pattern appears in two distinct regions: one with weakened transcriptional strength of miR-93 and the other with enhanced transcriptional strength of miR-93; for the other module (Figure 3(c), bottom right), the saturation pattern only appears in the region, in which the transcriptional strength of miR-93 is enhanced. Taken together, the results showed that for the two different network motifs the dynamical pattern of p21 is changing according to different combinations of its upstream regulators, suggesting the adaptation of p21 dynamics for different biological contexts.

**Figure 4:**
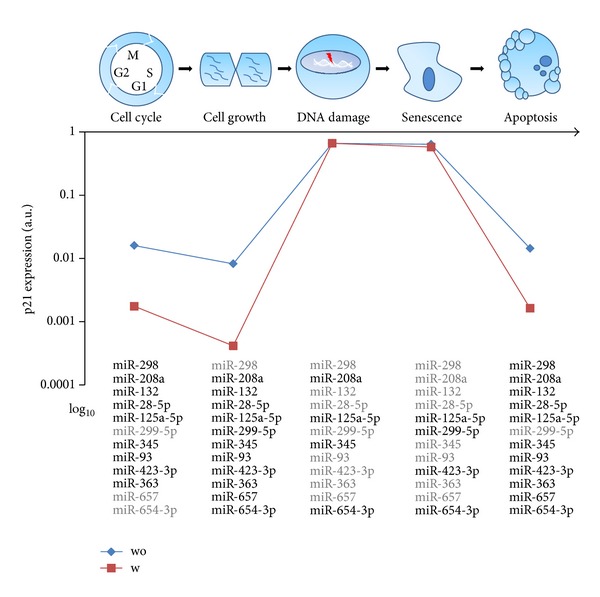
p21 expression regulated by cooperative miRNAs for different cellular processes. The associations of the miRNAs with these cellular processes were derived from GO terms of their TFs. A miRNA was supposed to be expressed (in bold black font) in a cellular process only if its TF is related to the corresponding GO term of this process. The p21 expression levels are computed for each process with (w)/without (wo) considering the cooperative effect among the p21 targeting miRNAs.

Furthermore, we performed a number of simulations to show the influence of miRNA regulation on p21 expression levels in different cellular processes arranged in a consecutive manner. In this procedure, a cell is first in the process of cell cycle followed by proliferation (cell growth), then the cell responses to DNA damage and enters into the process of senescence, and finally apoptosis is initiated. As shown in Figure 4, during the cell cycle process, the p21 expression is low due to the activation of most of its targeting miRNAs; when the cell starts proliferating, the p21 expression declines to an even lower level because of more activated miRNAs in this process; after responding to DNA damage, the p21 expression soars to a high level, which is caused by the activation of its TFs like p53 and fewer expressed miRNAs.; although the p21 expression keeps at a similar level while the cell is undergoing senescence, the expressed miRNAs are different from the previous process; finally, the p21 expression decreases again to a low level due to the reemergence of most of its targeting miRNAs and the cell enters apoptosis. Interestingly, the model simulations are also consistent with experimental observations: under non-stressed condition the low expression level of p21 is needed for cell proliferation; the upregulation of p21 happens after response to DNA damage via p53 and the increased p21 expression further results in cell cycle arrest leading to senescence and apoptosis [25]. Besides, when considering the effect of cooperating miRNAs, the p21 expression levels were indistinguishable from the previous simulation of DNA damage response. However, for the other processes p21 expression levels were significantly lower compared to the simulations without considering the cooperative effect of the miRNAs. Above all, these results indicated that selective expression of cooperative miRNAs could be adopted by cells to ensure diverse expression levels of p21 to meet the requirements of different cellular processes.

## 3. Conclusions and Discussion

In this paper, we presented a systems biology approach, combining data-driven modeling and model-driven experiments, to investigate the role of miRNA-mediated repression in gene regulatory networks. This approach provides a systematic way to gain a deeper understanding of the regulation of target genes by mutiple and cooperative miRNAs. Using the regulation of p21 by multiple miRNAs as a case study, we showed how the ODE-based model, which is calibrated and validated by means of experimental data, is suitable for predicting the temporal dynamics of molecular concentrations involved in biochemical systems. 

Provided there are sufficiently rich quantitative data sets avaliable to characterize the model, the use of the methodology here shown can be extended to more complex regulatory networks, involving multiple targets, cooperating TFs and miRNAs and signaling pathways displaying cross-talk via post-tranlational modifications. In this case, the critical element is the quality and quantitiy of the available data. Insufficiency and low quality of experimental data can cause errors in the process of model construction and overfitting in parameter estimation can lead to uncertainties in the model predictions. We believe that the quick development of quantitative high throughput techniques such as transcriptomics, proteomics and miRNomics will facilitate the construction and characterization of larger miRNA-mediated regulatory networks.

Other modeling frameworks than ODE-based models can be used to describe biological systems, such as probabilistic (e.g., Bayesian) or logical (e.g., Boolean) models. Importantly, different modeling frameworks have different properties and perform well regarding different perspectives and levels of mechanistic details of biochemical systems [46]. For example, Bayesian models are helpful in the construction of connections in signaling networks and can reveal the most likely underlying structure of the network in a probabilistic manner. Boolean models use binary values (0 and 1) and logical gates (AND, NOT, and OR) to describe activities of network components and the information flow among them. We believe that in the coming future, hybrid models, which consist of modeling framework and experimental technique specific sub-modules, will provide the necessary compromise between quantitative/qualitative accuracy and scalability for the investigation of large biochemical networks [47]. 

## Figures and Tables

**Table 1:** Overview of the methodology. Key points in each step of the methodology and the main resources for constructing miRNA-mediated gene regulatory networks are given.

Step 1: data retrieval	
Regulation types	Resources
Transcriptional gene regulation	TRED (http://rulai.cshl.edu/cgi-bin/TRED/tred.cgi?process=home): a database that provides an integrated repository for both cis- and transregulatory elements in mammals TRANSFAC (http://www.gene-regulation.com/pub/databases.html): a database that collects eukaryotic transcriptional regulation, comprising data on TFs, their target genes, and binding sites The UCSC table browser (http://genome.ucsc.edu/): a popular web-based tool for querying the UCSC Genome Browser annotation tables HTRIdb (http://www.lbbc.ibb.unesp.br/htri/): an open-access database for experimentally verified human transcriptional regulation interactions MIR@NT@N (http://maia.uni.lu/mironton.php/): an integrative resource based on a metaregulation network model including TFs, miRNAs, and genes PuTmiR (http://www.isical.ac.in/~bioinfo_miu/TF-miRNA.php): a database of predicted TFs for human miRNAs TransmiR (http://202.38.126.151/hmdd/mirna/tf/): a database of validated TF-miRNA interactions miRGen 2.0 (http://diana.cslab.ece.ntua.gr/mirgen/): a database of miRNA genomic information and regulation

Posttranscriptional gene regulation	miRecords (http://mirecords.biolead.org/): a resource for animal miRNA-target interactions Tarbase (http://www.microrna.gr/tarbase/): a database that stores detailed information for each miRNA-gene interaction, the experimental validation methodologies, and their outcomes miRTarBase (http://mirtarbase.mbc.nctu.edu.tw/): a database that collects validated miRNA-target interactions by manually surveying the pertinent literature miRWalk (http://www.umm.uni-heidelberg.de/apps/zmf/mirwalk/): a comprehensive database that provides information on miRNAs from human, mouse, and rat, on their predicted as well as validated binding sites in target genes

Protein-protein interaction	HPRD (http://www.hprd.org/): a centralized platform to visually depict and integrate information pertaining to domain architecture, posttranslational modifications, interaction networks, and disease association for each protein in the human proteome STRING (http://string-db.org/): a database of known and predicted protein interactions. The interactions include direct (physical) and indirect (functional) associations MPPI (http://mips.helmholtz-muenchen.de/proj/ppi/): a collection of manually curated high-quality PPI data collected from the scientific literature by expert curators DIP (http://dip.doe-mbi.ucla.edu/dip/Main.cgi): a catalog of experimentally determined interactions between proteins IntAct (http://www.ebi.ac.uk/intact/main.xhtml): a platform that provides a database system and analysis tools for molecular interaction data Reactome (http://www.reactome.org/): an open-source, open access, manually curated, and peer-reviewed pathway database

GO annotation	Amigo GO (http://amigo.geneontology.org/cgi-bin/amigo/go.cgi): the official GO browser and search engine miR2Disease (http://www.mir2disease.org/): a manually curated database that aims at providing a comprehensive resource of miRNA deregulation in various human diseases miRCancer (http://mircancer.ecu.edu): a miRNA-cancer association database constructed by text mining on the literature PhenomiR (http://mips.helmholtz-muenchen.de/phenomir/): a database that provides information about differentially expressed miRNAs in diseases and other biological processes miRGator (http://mirgator.kobic.re.kr/): a novel database and navigator tool for functional interpretation of miRNAs miRó (http://ferrolab.dmi.unict.it/miro): a web-based knowledge base that provides users with miRNA-phenotype associations in humans

Step 2: network construction and visualization	
(i) Visualize regulatory interactions in platforms such as CellDesigner and Cytoscape that support standardized data formats	
(ii) Calculate confidence scores for assessing reliability of interactions in gene regulatory networks	

Step 3: model construction and calibration	
(i) Formulate equations using rate equations	
(ii) Fix parameter values using available biological information	
(iii) Estimate the other unknown and immeasurable parameter values using optimization methods which can minimize the distance between model simulations and experimental data such as time course qRT-PCR and western blot data	

Step 4: model validation and analysis	
(i) Design new experiments and generate new data to verify the calibrated model	
(ii) Study complex properties and behavior of the system	

**Table:** (a) miRNA-p21 interaction scores

miRNA	miR-125a-5p	miR-132	miR-208a	miR-28-5p	miR-298	miR-299-5p	miR-345	miR-363
Score	0.73	0.73	0.73	0.80	0.73	0.80	0.73	0.73

miRNA	miR-423-3p	miR-515-3p	miR-572	miR-639	miR-654-3p	miR-657	miR-93	
Score	0.85	0.73	0.73	0.73	0.73	0.80	0.95	

**Table:** (b) TF-miRNA interaction scores

	Score		Score		Score		Score		Score		Score
miR-208		miR-132		miR-657		miR-125a-5p		miR-28-5p		miR-93	
MAX	0.27	EGR1	0.93	TFAP2A	0.34	EGR1	0.86	MZF1	0.28	TFAP2A	0.34
EGR1	0.29	CREB1	0.86	SP1	0.34	PAX5	0.24	E47	0.27	SOX9	0.24
ARNT	0.27	AREB6	0.27	MZF1	0.34	NRSF	0.24	FOXC1	0.31	GATA3	0.27
RFX1	0.24	E47	0.24	MAX	0.29	ZID	0.24	FOXI1	0.24	MZF1	0.31
ATF6	0.24	ELK1	0.27	USF1	0.27	PPARG	0.27	SRY	0.27	SP1	0.34
YY1	0.27	EGR2	0.29	EGR1	0.34	GATA3	0.34	JUN	0.29	POU2F1	0.24
JUNB	0.24	FOS	0.24	RELA	0.29	MZF1	0.34	POU2F1	0.24	EGR1	0.29
POU2F1	0.24	NFIC	0.24	miR-654-3p		TP53	0.34	SRF	0.27	CEBPA	0.24
NFIC	0.27	RELA	0.27	YY1	0.27	NFIC	0.34	CEBPA	0.29	NFYA	0.24
miR-345		miR-423-3p		BACH1	0.24	miR-299-5p		RORA	0.24	RUNX1	0.24
SP1	0.34	BACH1	0.27	FOXL1	0.29	CREB1	0.24	miR-363		STAT1	0.24
RELA	0.27	STAT1	0.27	SRY	0.24	SRY	0.27	E2F1	0.62	E2F1	0.85
NFATC2	0.24	POU3F2	0.24	NFATC2	0.24	SRF	0.29	FOXC1	0.33	MYC	0.84
HNF4A	0.31	STAT5A	0.27	FOS	0.24	RUNX1	0.27	MZF1	0.34	miR-298	
NFYA	0.24	SRF	0.27	POU2F1	0.27	miR-572		miR-639		JUNB	0.31
POU2F1	0.24	MEF2A	0.27	NF*κ*B1	0.24	FOXF2	0.24	NFYA	0.24		
NFIC	0.31	POU2F1	0.24	HNF1A	0.24						

**Table:** (c) TF-p21 interaction scores

TF (verified)	SP1	SP3	E2F1	Runx1	Runx2	STAT1	E2A	STAT5	TP53	TP63	TP73
Score	1.00	1.00	0.77	0.77	0.77	0.72	0.72	0.67	0.67	0.67	0.67

TF (verified)	STAT3	CUX1	TFAP2A	BRCA1	VDR	RARA	C/EBP*α*	C/EBP*β*	RB1	Tbx2	EHMT2
Score	0.66	0.66	0.60	0.60	0.60	0.60	0.60	0.60	0.60	0.60	0.60

TF (putative)	NF*κ*B1	RELA	STAT2	STAT6	SRF						
Score	0.44	0.44	0.44	0.44	0.43						

**Table:** (d) p21-protein interaction scores

Protein	TP53	PCNA	CASP3	CCNA1	CCND1	SKP2	BCCIP	CCNA2	CCND2	CCNE2	AKT1	C1orf123	CCDC85B	CCNB1	CCNB2	CCND3
Score	1.00	0.92	0.87	0.87	0.87	0.87	0.81	0.81	0.81	0.81	0.72	0.72	0.72	0.72	0.72	0.72

Protein	CCNE1	CDC45	CDC5L	CDC6	CDC7	CDK1	CDK14	CDK2	CDK3	CDK4	CDK6	CEBPA	CIZ1	CSNK2A1	CSNK2B	DAPK3
Score	0.72	0.72	0.72	0.72	0.72	0.72	0.72	0.72	0.72	0.72	0.72	0.72	0.72	0.72	0.72	0.72

Protein	ESR1	GADD45A	GADD45B	GADD45G	GMNN	GNB2L1	HDAC11	ITGB1BP3	MAP3K5	MAPK8	MCM10	PARP1	PIM1	POLD2	PSMA3	RAB1A
Score	0.72	0.72	0.72	0.72	0.72	0.72	0.72	0.72	0.72	0.72	0.72	0.72	0.72	0.72	0.72	0.72

Protein	SET	SLC25A11	STAT3	TEX11	TK1	TSG101	TTLL5	XRCC6	CDC20	CDC27	CDK5	DHX9	MDM2	NR4A1	PRKCH	RPA1
Score	0.72	0.72	0.72	0.72	0.72	0.72	0.72	0.72	0.56	0.56	0.56	0.56	0.56	0.56	0.56	0.56

Verified: experimentally verified interaction; putative: predicted interaction.

**Table 3:** Initial concentrations of model variables and model parameter values. Based on the experimental data, the p21-targeting miRNAs verified by Wu et al. [23] were divided into two groups: the translation repression group (marked with asterisk) and the mRNA deadenylation group. A miRNA was classified into the mRNA deadenylation group if its overexpression can result in 20% or more downregulation of the p21 mRNA level (i.e., p21 mRNA level ≤ 0.8; the basal level is 1); otherwise, it was classified into the translation repression group. For the translation repression group, only *k*_ass_^complex_*i*_^ was estimated and *k*_deg⁡_^complex_*i*_^ was fixed. For the other group, both *k*_deg⁡_^complex_*i*_^and *k*_ass_^complex_*i*_^ were estimated. The initial concentrations of p21 and mp21 were set to 1, and this value was used as their basal expression levels. During the parameter estimation, the initial concentrations of p21-targeting miRNAs were set to 100, because in the publication [23] the expression levels of p21  and mp21 were measured after the individual introduction of the p21-targeting miRNAs with amount of 100 nM. Due to the lack of biological information to characterize the transcriptional activation function (*f*_act_) of p21 and its targeting miRNAs, the corresponding functions were assumed to be 1 for simplicity. The data is adapted from our previous publication [4].

Initial concentration of variables and TF functions					
Variable	Description			Initial concentration (a.u.)	
p21	p21 protein			1	
mp21	p21 mRNA			1	
miR_*i*=1,…,15_	p21-targeting miRNAs			100	
[mp21 | miR_*i*=1,…,15_]	Complexes formed by miR_*i*_ and mp21			0	
*f*_act_(TF_mp21_)	p21's transcriptional activation function			1	
*f*_act_ (TF_miR_*i* (*i*=1,…,15)__)	The transcriptional activation function of miR_*i*_			1	

Fixed parameter values					
Parameter	Description			Value (hr^−1^)	Reference

*k*_syn_^mp21^	Synthesis rate constant of mp21			0.1155	fixed
*k*_deg⁡_^mp21^	Degradation rate constant of mp21			0.1155	[24]
*k*_syn_^miR_*i*_^ (*i* = 1,…, 15)	Synthesis rate constant of miR_*i*_			0.0289	fixed
*k*_deg⁡_^miR_*i*_^ (*i* = 1,…, 15)	Degradation rate constant of miR_*i*_			0.0289	[25]
*k*_syn_^p21^	Synthesis rate constant of p21			1.3863	fixed
*k*_deg⁡_^p21^	Degradation rate constant of p21			1.3863	[26]

Estimated parameter values					
miRNA (state variable)	*k*_deg⁡_^complex_*i*_^ (*i* = 1,…, 15) (hr^−1^)	*k*_ass_^complex_*i*_^ (*i* = 1,…, 15) (a.u. ^−1^ *·* hr^−1^)	*F*_cost_^miR_*i*_^ (*i* = 1,…, 15)	Experimental data of p21 (protein, mRNA ± SD)	

miR-298 (miR_1_)*	0.1155	0.0254	3.4*e* − 004	(0.16, 1.074 ± 0.025)	
miR-208a (miR_2_)*	0.1155	0.0041	2.0*e* − 003	(0.51, 1.192 ± 0.022)	
miR-132 (miR_3_)*	0.1155	0.0275	2.4*e* − 003	(0.15, 1.21 ± 0.147)	
miR-28-5p (miR_4_)*	0.1155	0.0119	5.9*e* − 003	(0.28, 1.35 ± 0.06)	
miR-125-5p (miR_5_)*	0.1155	0.0018	1.8*e* − 003	(0.69, 0.85 ± 0.051)	
miR-299-5p (miR_6_)*	0.1155	0.0080	1.8*e* − 004	(0.36, 0.95 ± 0.038)	
miR-345 (miR_7_)*	0.1155	0.0051	1.1*e* − 004	(0.46, 0.96 ± 0.039)	

miR-93 (miR_8_)	0.1564	0.0235	4.1*e* − 014	(0.17, 0.7776 ± 0.03)	
miR-423-3p (miR_9_)	0.9118	0.0055	2.8*e* − 009	(0.44, 0.5102 ± 0.11)	
miR-515-3p (miR_10_)	0.2098	0.0253	1.2*e* − 013	(0.16, 0.616 ± 0.037)	
miR-363 (miR_11_)	0.2261	0.0399	2.2*e* − 014	(0.11, 0.56 ± 0.15)	
mR-657 (miR_12_)	0.3465	0.0158	2.1*e* − 014	(0.23, 0.48 ± 0.12)	
miR-639 (miR_13_)	0.4305	0.0327	1.8*e* − 017	(0.13, 0.36 ± 0.084)	
miR-572 (miR_14_)	0.3039	0.0360	9.4*e* − 023	(0.12, 0.45 ± 0.044)	
miR-654-3p (miR_15_)	9.7485	0.0024	3.0*e* − 014	(0.64, 0.63 ± 0.053)	
